# Supplementary material for: A Comparison of the Stability of Refined Edible Vegetable Oils under Frying Conditions: Multivariate Fingerprinting Approach
Source: Foods. 2023 Feb 1;12(3):604. doi: 10.3390/foods12030604 (PMC9914197; doi:10.3390/foods12030604)
Supplement: Supplementary file 1 [file foods-12-00604-s001.zip › foods-2150926-supplementary.pdf]

foods

Supplementary material

A Comparison of the Stability of Refined Edible Vegetable Oils under Frying Conditions: Multivariate Fingerprinting Approach

Sandra Martín-Torres, Antonio González-Casado, Miriam Medina-García, María. Soledad Medina-Vázquez and Luis Cuadros-Rodríguez *

Department of Analytical Chemistry, Faculty of Sciences, University of Granada, Av. Fuentenueva s.n., E-18071 Granada, Spain
* Correspondence: lcuadros@ugr.es

Table S1. Experimental data value matrix (106 × 7): %TPC: total polar content; RI: refractive index; PV: peroxide value; AV: anisidine value; K232: absorptivity at 232 nm; K268: absorptivity at 268 nm.

Sample code	% TPC	RI	PV	AV	K232	K268
ORUIFA00	0.0	1.471	6.1	3.2	3.5	1.7
ORUIFA02	2.0	1.473	9.2	33.2	5.1	1.9
ORUIFA04	5.0	1.472	8.8	59.4	6.4	2.5
ORUIFA06	7.5	1.472	7.7	73.0	7.6	2.8
ORUIFA08	10.5	1.472	8.3	81.5	8.9	2.8
ORUIFA10	12.0	1.473	8.5	93.6	10.1	2.7
ORUIFA12	14.5	1.475	6.7	95.1	10.7	2.8
ORUIFA14	19.5	1.475	8.4	99.9	11.7	2.8
ORUIFA16	23.5	1.475	10.4	105.3	12.4	2.8
ORUIFA18	24.5	1.475	10.3	111.8	13.0	2.8
ORUIFA20	30.0	1.476	11.9	112.4	13.4	2.9
ORUDIA00	0.0	1.470	3.7	1.9	4.1	1.5
ORUDIA02	3.5	1.472	6.3	25.6	5.4	2.3
ORUDIA04	6.0	1.471	7.1	43.7	6.6	3.0
ORUDIA06	8.5	1.472	5.9	62.1	7.9	3.5
ORUDIA08	11.5	1.472	6.2	69.2	8.9	3.7
ORUDIA10	16.0	1.472	9.0	72.9	9.9	3.7
ORUDIA12	17.0	1.473	5.1	75.9	10.8	3.6
ORUDIA14	19.5	1.473	4.1	87.8	12.2	3.7
ORUDIA16	22.0	1.473	4.8	95.8	12.6	3.7
ORUDIA18	25.5	1.472	5.5	96.2	13.8	3.7
ORUDIA20	30.5	1.472	4.4	97.6	13.9	3.6
OLIHAC00	0.0	1.468	8.3	3.6	2.0	0.6
OLIHAC02	0.0	1.469	11.5	27.8	3.5	1.3
OLIHAC04	0.0	1.469	13.5	46.9	4.6	1.4
OLIHAC06	0.5	1.469	15.7	56.9	6.0	1.5
OLIHAC08	6.5	1.469	13.4	66.5	7.1	1.4
OLIHAC10	10.5	1.470	18.4	73.2	7.8	1.2
OLIHAC12	10.0	1.470	21.3	78.8	8.9	1.4
OLIHAC14	14.0	1.470	18.9	83.2	9.6	1.3
OLIHAC16	15.5	1.470	24.6	75.5	10.3	1.3
OLIHAC18	21.0	1.470	22.8	83.5	11.0	1.4
OLIHAC20	27.0	1.470	15.9	86.2	11.5	1.5
OLIHAC22	29.0	1.471	16.0	87.4	12.1	1.8
OLIESP00	0.0	1.468	9.3	1.8	2.2	0.8
OLIESP02	0.0	1.469	11.1	26.7	3.7	1.3

OLIESP04	0.5	1.469	8.3	34.5	4.2	1.4
OLIESP06	2.0	1.469	9.3	47.1	4.9	1.4
OLIESP08	5.0	1.469	10.3	52.8	6.4	1.6
OLIESP10	3.5	1.470	9.0	59.6	7.1	4.2
OLIESP12	8.0	1.470	10.4	65.6	8.5	1.5
OLIESP14	8.5	1.470	10.5	69.6	8.9	1.4
OLIESP16	13.0	1.470	10.5	74.6	9.9	1.5
OLIESP18	15.0	1.470	11.0	79.5	10.7	1.5
OLIESP20	19.0	1.470	31.6	77.1	11.5	1.5
OLIESP22	22.0	1.470	11.0	87.8	12.1	1.9
OLIESP24	26.5	1.471	10.3	88.5	12.5	1.7
OLIESP26	28.0	1.471	9.2	96.6	13.2	1.8
GIRBOR00	0.5	1.476	12.6	6.4	3.3	1.2
GIRBOR02	3.5	1.476	17.5	56.2	7.8	2.3
GIRBOR04	6.0	1.475	26.3	97.3	11.4	3.3
GIRBOR06	8.0	1.476	23.2	132.2	15.2	4.2
GIRBOR08	14.5	1.476	21.3	158.5	17.1	4.8
GIRBOR10	15.5	1.477	16.5	200.6	19.8	5.4
GIRBOR12	20.0	1.477	15.3	176.6	21.8	5.7
GIRBOR14	27.0	1.477	13.0	186.7	23.5	5.9
GIRBOR16	26.0	1.477	13.3	203.9	25.2	6.2
GIRBOR18	29.5	1.478	15.4	225.7	26.9	6.2
GIRCOO00	0.5	1.473	4.6	5.6	2.8	1.0
GIRCOO04	6.0	1.475	5.7	32.9	5.4	1.9
GIRCOO08	5.5	1.475	8.2	48.4	8.1	2.7
GIRCOO12	7.5	1.475	8.7	66.0	10.1	3.5
GIRCOO16	9.0	1.477	8.8	77.5	11.9	4.1
GIRCOO20	8.0	1.475	11.7	118.5	13.8	4.5
GIRCOO24	9.5	1.478	13.4	119.3	15.7	4.9
GIRCOO28	13.0	1.476	11.0	122.9	17.1	5.3
GIRCOO32	14.5	1.475	14.2	131.2	18.5	5.3
GIRHAC00	0.0	1.472	5.4	6.6	3.2	1.4
GIRHAC04	2.5	1.473	4.2	28.3	4.9	1.8
GIRHAC08	1.5	1.473	7.9	42.4	6.8	2.2
GIRHAC12	3.5	1.473	8.2	50.0	9.2	2.8
GIRHAC16	4.0	1.473	8.7	63.0	9.6	2.9
GIRHAC20	6.5	1.473	6.9	77.2	10.9	3.2
GIRHAC24	8.5	1.473	8.5	85.8	12.8	3.7
GIRHAC28	7.0	1.473	6.3	89.8	13.8	3.9
GIRHAC32	9.0	1.473	5.7	96.9	14.6	4.0
SEMIFA00	0.0	1.471	9.8	1.9	2.1	1.0
SEMIFA02	0.0	1.471	8.7	45.7	3.1	1.3
SEMIFA04	0.5	1.471	8.9	70.4	4.9	1.4
SEMIFA06	3.0	1.471	7.5	89.2	11.7	1.5
SEMIFA08	6.0	1.471	6.0	102.2	13.5	1.4
SEMIFA10	9.5	1.471	7.2	112.5	11.4	1.2
SEMIFA12	11.0	1.471	9.2	114.9	14.9	1.4
SEMIFA14	17.0	1.471	7.4	118.1	14.0	1.3
SEMIFA16	18.0	1.471	10.0	122.0	14.3	1.3
SEMIFA18	20.0	1.471	10.3	131.1	14.1	1.4
SEMIFA20	26.5	1.472	8.0	124.0	11.8	1.5
SEMIFA22	31.0	1.472	7.4	140.4	13.9	1.8
GIRKOI00	0.0	1.473	3.0	3.0	3.8	1.8
GIRKOI04	2.5	1.474	3.1	19.4	5.4	2.1
GIRKOI08	4.0	1.474	4.0	32.0	7.2	2.4
GIRKOI12	5.5	1.474	4.0	42.0	8.9	2.7
GIRKOI16	7.5	1.475	6.5	53.2	10.7	3.1

GIRKOI20	9.5	1.475	9.8	65.8	16.8	4.7
GIRKOI24	12.0	1.476	6.6	72.1	14.1	3.8
GIRKOI28	12.5	1.476	7.2	76.8	16.0	4.3
GIRKOI32	13.5	1.478	8.5	83.0	17.6	4.2
SEMHAC00	0.0	1.471	1.1	4.7	6.1	2.6
SEMHAC04	0.0	1.472	4.4	18.5	9.6	4.7
SEMHAC08	0.5	1.472	4.3	28.1	11.4	4.6
SEMHAC12	1.0	1.471	2.7	35.3	14.8	5.9
SEMHAC16	0.5	1.472	4.4	53.1	16.9	6.1
SEMHAC20	2.5	1.472	4.6	59.1	20.5	7.3
SEMHAC24	3.0	1.473	6.1	63.5	24.3	8.8
SEMHAC28	1.0	1.472	5.2	59.9	25.4	8.6
SEMHAC32	4.5	1.472	4.3	56.2	27.5	9.0
